# Toll-like receptor 4 signaling promotes epithelial-mesenchymal transition in human hepatocellular carcinoma induced by lipopolysaccharide

**DOI:** 10.1186/1741-7015-10-98

**Published:** 2012-08-31

**Authors:** Ying-Ying Jing, Zhi-Peng Han, Kai Sun, Shan-Shan Zhang, Jing Hou, Yan Liu, Rong Li, Lu Gao, Xue Zhao, Qiu-Dong Zhao, Meng-Chao Wu, Li-Xin Wei

**Affiliations:** 1 Tumor Immunology and Gene Therapy Center, Eastern Hepatobiliary Surgery Hospital, Second Military Medical University, Shanghai, PR China

**Keywords:** Toll-like receptor 4, Epithelial-mesenchymal transition, Lipopolysaccharide, Human hepatocellular carcinoma

## Abstract

**Background:**

The endotoxin level in the portal and peripheral veins of hepatocellular carcinoma (HCC) patients is higher and lipopolysaccharide (LPS), a cell wall constituent of gram-negative bacteria, has been reported to inhibit tumor growth. However, in this study, we found that LPS-induced toll-like receptor 4 (TLR4) signaling was involved in tumor invasion and survival, and the molecular mechanism was investigated,

**Methods:**

Four HCC cell lines and a splenic vein metastasis of the nude mouse model were used to study the invasion ability of LPS-induced HCC cells and the epithelia-mesenchymal transition (EMT) *in vitro *and *in vivo*. A total of 106 clinical samples from HCC patients were used to evaluate TLR4 expression and analyze its association with clinicopathological characteristics

**Results:**

The *in vitro *and *in vivo *experiments demonstrated that LPS could significantly enhance the invasive potential and induce EMT in HCC cells with TLR4 dependent. Further studies showed that LPS could directly activate nuclear factor kappa B (NF-κB) signaling through TLR4 in HCC cells. Interestingly, blocking NF-κB signaling significantly inhibited transcription factor Snail expression and thereby inhibited EMT occurrence. High expression of TLR4 in HCC tissues was strongly associated with both poor cancer-free survival and overall survival in patients.

**Conclusions:**

Our results indicate that TLR4 signaling is required for LPS-induced EMT, tumor cell invasion and metastasis, which provide molecular insights for LPS-related pathogenesis and a basis for developing new strategies against metastasis in HCC.

## Background

Persistent inflammatory conditions can induce tumorigenesis [1]. Hepatocellular carcinoma (HCC) is closely associated with chronic inflammatory liver diseases and the endotoxin level in the portal and peripheral veins of those patients is higher owing to changes in the intestinal mucosal permeability and increased bacterial infection [2-4]. Lipopolysaccharide (LPS), a cell wall constituent of gram-negative bacteria, is released during lysis of bacteria. It has been reported that LPS can induce cytokines from immune cells and inhibit tumor growth [5,6], but recent studies have shown that LPS can alter cytokine levels in the tumor microenvironment and exert direct effects on tumor cell proliferation, invasion and metastasis *in vitro *and *in vivo *[7-10].

Toll-like receptor 4 (TLR4), the receptor for LPS, is not only important in the regulation of immune responses to infection [11], but also is involved in noninfectious inflammatory diseases, such as tumor invasion and survival [12]. TLR4 has been detected in many human cancer cell lines, including pancreatic, lung, breast, prostate, liver and colorectal cancer [10,12-16]. When silencing TLR4 expression, the invasion, survival, and tumorigenicity of human prostate cancer cells was inhibited, which indicates TLR4 plays a significant role in connecting inflammation and cancer invasion and progression [12]; however, the exact mechanism is still not clear.

Epithelial to mesenchymal transition (EMT) is a process in which epithelial cell layers lose polarity together with cell to cell contacts which results in a dramatic remodeling of the cytoskeleton, and has an important role in tumor metastasis [17]. When human intrahepatic biliary epithelial cells (IBECs) were exposed to high levels of LPS, the IBECs could undergo EMT, potentially contributing to hepatic fibrosis or even hepatoma [18]. In HCC tissues, LPS was previously reported to promote adhesion and invasion in hepatoma cells [9]. These effects suggest that LPS-induced TLR4 signaling provides a survival benefit for metastatic tumors; however, whether TLR4 signaling can induce EMT in HCC cells and the mechanism involved remains unclear.

In the present study, we provide evidence that LPS-induced TLR4 signaling promotes HCC cell invasion and EMT *in vitro *and *in vivo*, and a high expression of TLR4 in HCC tissues was strongly associated with both poor cancer-free survival and overall survival in patients, which indicates that LPS is closely related to tumor invasion and metastasis, rather than only anti-tumor effects.

## Methods

### Reagents and antibodies

(Dulbecco's) modified Eagle's medium ((D)MEM), fetal bovine serum (FBS), penicillin, streptomycin sulfate, glutamine, and 0.05% trypsin/0.02% ethylenediamine tetraacetic acid (EDTA) solution were purchased from Invitrogen (Carlsbad, CA, USA). LPS derived from *Escherichia coli *strain 055:B5, Trizol, Lipofectamine 2000 and BAY11-7028, the inhibitor of nuclear factor kappa B (NF-κB), were purchased from Sigma (St. Louis, MO, USA). A dual luciferase reporter gene assay kit to detect NF-κB activity was purchased from Promega (Madison, WI, USA). Rabbit anti-human E-cadherin and Vimentin antibodies were obtained from Thermo (Fremont, CA, USA). Goat anti-human Snail antibody was purchased from R&D (Minneapolis, MN, USA). Rabbit anti-human TLR4 antibody was purchased from Bioworld Technology (St. Louis, MN, USA).

### Cell lines and culture condition

HCC cell lines (HepG2, Huh7, Hep3B, SMMC-7721 and MHCC97-H) were cultured in (D)MEM supplemented with 10% FBS, 100 U/ml penicillin, 100 μg/ml streptomycin sulfate, and 2.0 mM glutamine. Cells were maintained at 37°C in a humidified 5% CO_2 _atmosphere and subcultured by trypsinization with 0.05% trypsin/0.02% EDTA when cells became confluent. For LPS treatment, subconfluent cultures of cells were treated with 10 μg/ml LPS for 48 hours. For NF-κB inhibition, 10 μM BAY11-7028 was added to the cell culture at the same time as LPS.

### Patients and tissue specimens

Specimens of HCC tissues were obtained from 106 HCC patients who underwent hepatic resection at the Eastern Hepatobiliary Surgery Hospital of the Second Military Medical University from October 2000 to November 2003. These patients included 88 men and 18 women with a median age of 49 years (range: 11 to 72), and all of the specimens were subjected to immunohistochemisty (IHC). Prior informed consent was obtained and the study protocol was approved by the Ethics Committee of the Eastern Hepatobiliary Surgery Hospital.

### Wound healing and Transwell assay

The methods for wound healing and the Transwell assay have been described [19-21]. For the wound-healing assay, cells (5 × 10^4^) were seeded on a 24-well dish and incubated for 24 hours, the monolayer was then disrupted with a cell scraper (1.2 mm width), and photographs were taken at 0 and 48 hours in a phase-contrast microscope. Experiments were carried out in triplicate, and four fields of each point were recorded. For the Transwell assay, Boyden chambers (8 μm pore size) were coated with 200 μl Matrigel at 200 μg/ml and incubated overnight. Cells (5 × 10^4^) in medium without serum were plated in the upper chamber, and the medium containing 5% FBS was added in the lower chamber as a chemoattractant. After 24 hours of incubation at 37°C, the cells were fixed in 4% formaldehyde and stained with crystal violet dye, and the cells that invaded through the pores to the lower surface of the filter were counted under a microscope. Three invasion chambers were used per condition. The values obtained were calculated by averaging the total number of cells from three filters.

### Nude mouse splenic vein metastasis assay

All procedures involving animals were performed in accordance with the institutional animal welfare guidelines of the Second Military Medical University. Cells were injected into the splenic vein of eight-week-old nude mice (BALB/c strain) at 1 × 10^6 ^cells/injection site. The mice were sacrificed after six weeks and the numbers of surface liver metastases were counted.

### Quantitative real-time polymerase chain reaction (qPCR)

Total RNA extraction, complementary DNA (cDNA) synthesis, and qPCR were performed as described [22]. The primer sequences used in qPCR are shown in Additional file 1 Table S1.

### Western-blot analysis

Total soluble protein extractions from cultivated cells and western-blot analysis were performed as described [22]. Antibodies used in western-blot experiments were specific for TLR4, Snail or E-cadherin.

### Immunofluorescence

About 10^4 ^cells were seeded on a 48-well dish. After 24 hours, the cells were washed twice with PBS, and fixed in 4% paraformaldehyde and 0.1% Triton × 100 in PBS buffer at 4°C for 30 minutes. After being washed with PBS, the cells were incubated with the blocking solution (10% goat serum in PBS), and then incubated overnight with the primary antibodies, washed with PBS, and finally incubated with secondary antibodies at 37°C for 2 hours. After being stained with DAPI/PI (4', 6-diamidino-2-phenylindole/ propidium iodide), all matched samples were photographed using an immunofluorescence microscope and identical exposure times.

### FACS Analysis

The expression of TLR4 on HCC cells was determined using indirect immunofluorescent staining. In this analysis, 20 μl of anti-TLR4 antibody were added to 100 μl of cell suspension (1 × 10^6 ^cells/ml) and incubated at 4°C for 30 minutes, and further stained with secondary fluorescein isothiocyanate (FITC)-conjugated antibody at 4°C for 30 minutes. FITC-conjugated isotype IgG antibody was used as a negative control. The stained cells were analyzed on a FACS flow cytometer (Becton Dickinson, San Jose, CA, USA).

### Generation of stable TLR4-expressed and knocked-down cell lines

The adeno-associated viral construct which directs the expression of TLR4 (pAAV-TLR4-IRES-GFP), siRNA against TLR4 (pAAV-siTLR4-IRES-GFP) and their control vectors were constructed and verified by DNA sequencing. The adeno-associated viral construct that expresses TLR4 or siRNA targeting TLR4 were co-transfected with adeno-associated viral packaging plasmids pAAV-RC, pAAV-helper into 293T cells by Lipofectamine 2000 according to the manufacturer's instructions. At 24 and 48 hours post-transfection, culture medium was collected to be incubated with target cells. At 48 hours post-infection, infected cells were harvested for gene and protein expression analysis or selected with G418 (400 μg/ml) for two weeks to establish stable clones.

### NF-κB luciferase reporter assays

NF-κB luciferase reporter assays were performed as described previously [23]. Briefly, cells were co-transfected with a pNF-κB-luc reporter construct and a renilla luciferase-expressing plasmid (internal control to normalize for transfection efficiency) using Lipofectamine 2000 according to the manufacturer's instructions. After treatment for 48 hours, firefly and renilla luciferase activities were assessed using a dual luciferase reporter gene assay kit. NF-κB transcriptional activity = relative light units of firefly luciferase/relative light units of renilla luciferase.

### IHC analysis

The sample processing and IHC procedures were performed as described [20] and the results were expressed as the percentage of tumor cells with a positive stain. Thereafter, the percentage of TLR4-positive tumor cells was scored on a scale of 0 to 4 (0: no staining; 1: ≤10%; 2: 11% to 30%; 3: 31% to 50%; 4: ≥50%). Furthermore, the expression level of TLR4 was divided into the following two groups according to score: low (0, 1, 2) and high (3, 4) [16].

### Statistical analysis

All data, expressed as mean ± the standard error of the mean (SEM), were from at least three separate experiments. Groups were compared by analysis of variance (ANOVA) with a posteriori contrast by least significant difference; or by Student t-test using the Microsoft Excel Analysis ToolPak (Microsoft, Redmond, WA). *P *< 0.05 was considered significant.

## Results

### LPS induces metastasis and invasion of HCC cells lines

**Figure 1:**
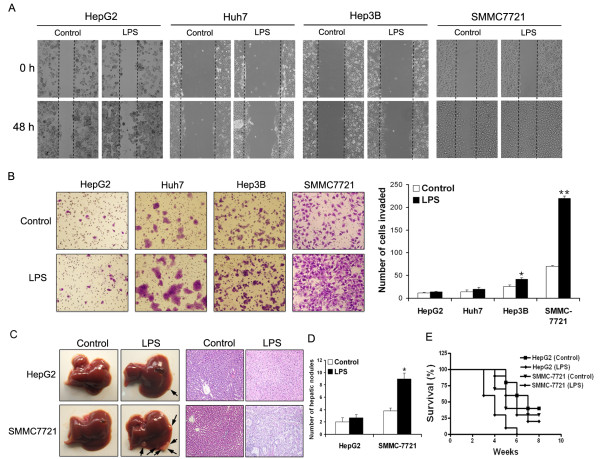
**Metastasis and invasion of HCC cells lines in response to LPS**. (**A**) The wound healing assay was employed to determine the migration of HepG2, Huh7, Hep3B and SMMC-7721 cells in response to LPS. Cells were monitored every 24 hours for two days to evaluate the rate of migration into the scratched area. (**B**) Invasiveness of cells was determined using the Transwell assay. Cells were treated with LPS (10 μg/ml) for 48 hours, and then plated in the upper chamber of the Transwell and allowed to grow for 24 hours in serum-free medium, 5% fetal bovine serum was placed in the lower chamber. The number of cells that invaded through the Matrigel was counted in ten fields under the ×20 objective lens, and is shown as the mean ± standard deviation (*P < 0.05, **P < 0.01). (A) and (B) are representative of at least three independent experiments (× 200). (**C**) Pictures of metastatic liver nodules in nude mice by splenic-vein injection of HepG2 and SMMC-7721 cells untreated or treated with LPS. H & E staining was performed on serial sections of metastatic tumors and normal liver (× 200). The arrows indicate the metastatic tumor on the surface of the liver. (**D**) The numbers of nodules were quantified on nude mice livers (n = 10 per group). Values for individual mice are shown above the bars (*P < 0.05). (**E**) Survival rate of nude mice six weeks after splenic vein injection of HepG2 and SMMC-7721 cells untreated or treated with LPS. HCC, hepatocellular carcinoma; LPS, lipopolysaccharide.

Four different human HCC cell lines (HepG2, Huh7, Hep3B, and SMMC-7721) were tested for the scratch wound-healing and Matrigel invasion assay. In HepG2 and Huh9 cells, no obvious effects on motility and invasion were observed in the presence of LPS stimulation (10 μg/ml) for 48 hours, and the effect in Hep3B cells was also limited. In contrast, the motility and invasion abilities of SMMC-7721 cells were enhanced significantly (Figure 1A, B, P < 0.05). We next chose HepG2, the least invasive cell line, and SMMC-7721, the most invasive one, for the splenic vein metastasis assay *in vivo*. Subconfluent monolayer cultures of cells were treated with or without LPS for 48 hours, and then were injected intrasplenically into athymic nude mice. After six to eight weeks, mice injected with LPS-treated or -untreated HepG2 cells had no difference in the number of liver surface metastatic nodules; however, the mice injected with LPS-treated SMMC-7721 cells had a significantly higher number of liver surface metastatic nodules compared to those with untreated cells (Figure 1C, D, P < 0.05). The survival rate of mice showed a similar trend (Figure 1E). These results indicate that LPS has diverse effects on the metastatic potential of HCC cell lines.

### LPS induces EMT in cells with higher expression of TLR4

**Figure 2:**
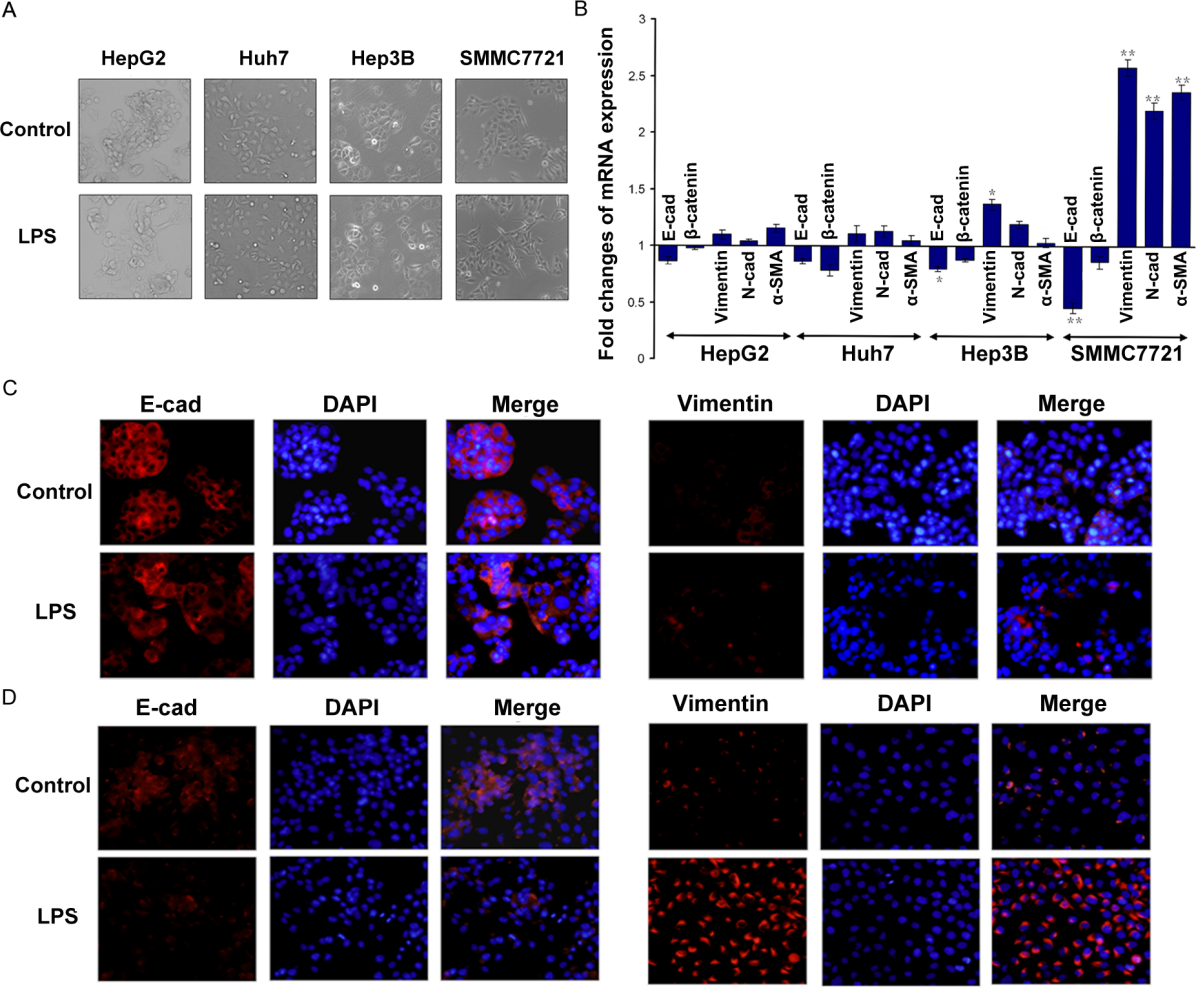
**LPS induces EMT in SMMC-7721 cells but not others**. (**A**) Phase-contrast photographs showing the morphology of HCC cells untreated or treated with LPS (10 μg/ml) for 48 h ours (× 200). (**B**) qPCR was used to detected changes in expression of EMT genes in HCC cells treated with LPS. Results presented represent mean of triplicate experiments ± SEM (*P < 0.05, **P < 0.01). (**C**) Immunofluorescent staining of E-cadherin and Vimentin was performed in HepG2 and SMMC-7721 cells that were either untreated or treated with LPS, nuclei were counterstained with DAPI (× 200). EMT, epithelial-mesenchymal transition; HCC, hepatocellular carcinoma; LPS, lipopolysaccharide; SEM, standard error of the mean.

Cell scattering was observed in SMMC-7721 cells treated with LPS, while no phenotypic changes were observed in the other cell lines (HepG2, Huh7 and Hep3B) (Figure 2A). Consistent with LPS promoting SMMC-7721 cell invasion, the mesenchymal markers (Vimentin, N-cadherin, and α-smooth muscle actin (α-SMA)) were upregulated and epithelial markers (E-cadherin and β-catenin) were downregulated. LPS did not induce EMT in HepG2, Huh7 and Hep3B cells (Figure 2B). The immunofluorescence results of E-cadherin and Vimentin further support the finding that LPS induced EMT in SMMC-7721 cells but not in HepG2 cells (Figure 2C).

### LPS-induced metastasis and EMT in HCC cells are dependent on TLR4

**Figure 3:**
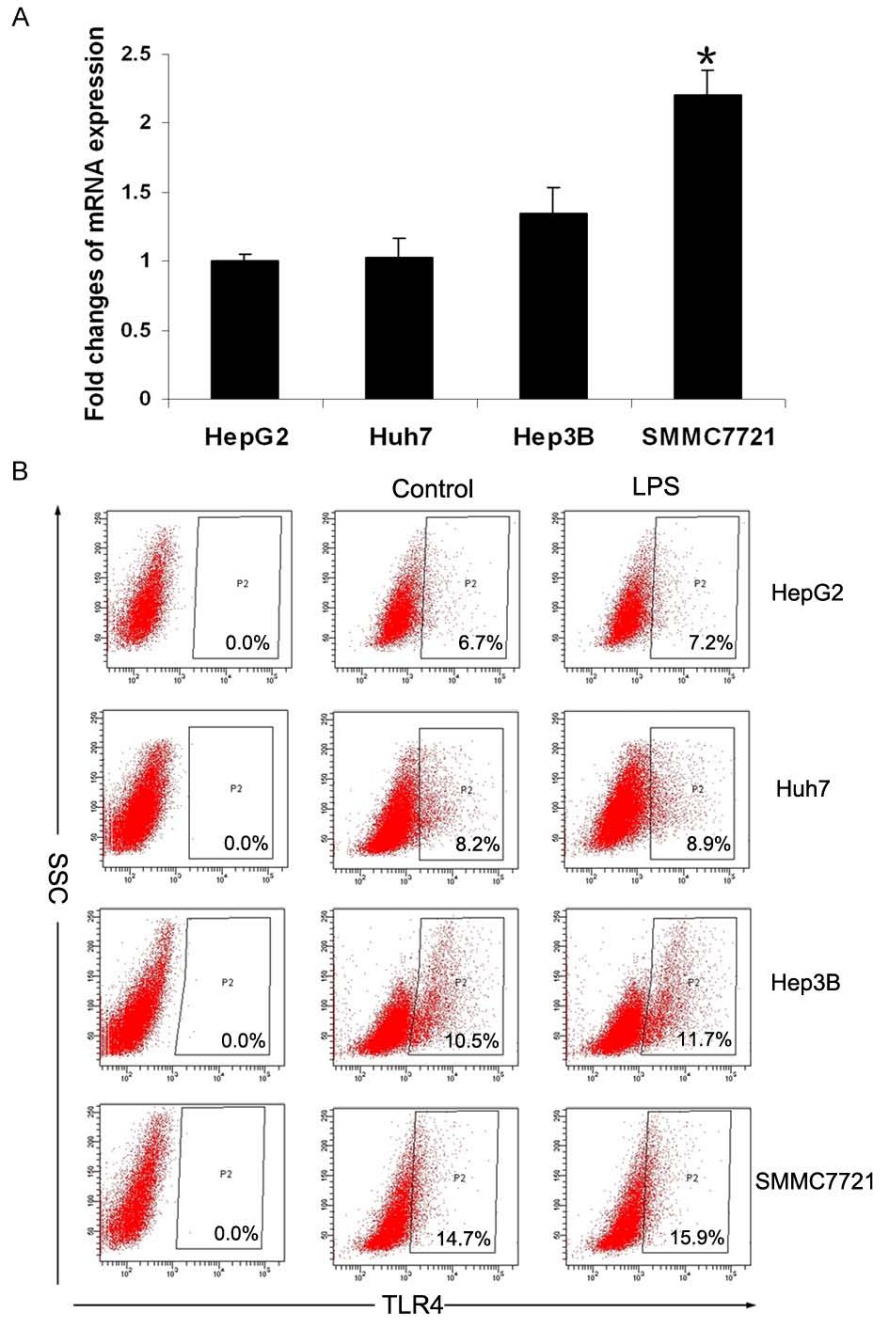
**TLR4 expression in different HCC cells lines**. (**A**) qPCR was used to detected TLR4 mRNA expression in HepG2, Huh7, Hep3B and SMMC-7721 cells (*P < 0.05). (**B**) FACS analysis for TLR4 protein in HCC cell lines untreated or treated with LPS (10 μg/ml) for 48 hours. The data shown in A and B are from one representative experiment of three performed. HCC, hepatocellular carcinoma; LPS, lipopolysaccharide; TLR4, toll-like receptor 4.

Based on real-time PCR analysis, the HCC cell line SMMC-7721 cells constitutively expressed a high level of TLR4, whereas Hep3B cells had low levels; on the other hand, the expression of TLR4 was almost absent in HepG2 and Huh7 cells (Figure 3A). Furthermore, FACS analysis showed that LPS had little effect on TLR4 expression (Figure 3B), suggesting that the different effects of LPS on HCC cell lines might be associated with intrinsic TLR4 expression. In addition, we detected TLR4 expression of the hepatocellular carcinoma cell line MHCC97-H cells which had been confirmed with a strong ability to metastasize. The results also showed that MHCC97-H cells expressed a high level of TLR4 which was not affected by LPS [See Additional file 1, Figure S1A]. LPS significantly promoted the invasion of the MHCC97-H cells through the Matrigel [See Additional file 1, Figure S1B]. The cell phenotype was also changed, which indicated that the mesenchymal markers (Vimentin and N-cadherin) were upregulated and the epithelial markers (E-cadherin and β-catenin) were downregulated [See Additional file 1, Figure S1C, D].

**Figure 4:**
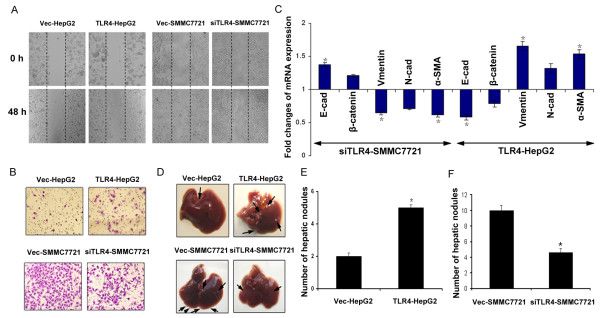
**Alteration of TLR4 influences LPS-induced metastasis and EMT of HCC cells**. (**A**) and (**B**) HepG2 cells were transduced with adeno-associated virus to express TLR4 and SMMC-7721 cells were transduced with siRNA to knocked down TLR4, and then the cells were treated with LPS. The migration of pretreated HCC cells was determined by the wound healing assay (A, × 200) and the invasiveness of pretreated HCC cells was determined by the Transwell assay (B, × 200). (**C**) Expression of EMT genes in pretreated HCC cells was detected by qPCR (normalized to β-actin) Results presented represent the mean of triplicate experiments ± SEM (*P < 0.05, **P < 0.01). (**D**) Pictures of metastatic liver nodules in nude mice by splenic-vein injection of pretreated HCC cells (× 200). The arrows indicate the metastatic tumor on the surface of the liver (n = 10 per group). (**E**) and (**F**) The number of nodules was quantified on nude mice livers by splenic-vein injection of pretreated HCC cells. Values for individual mice are shown above the bars (*P < 0.05). EMT, epithelial-mesenchymal transition; HCC, hepatocellular carcinoma; LPS, lipopolysaccharide; SEM, standard error of the mean; TLR4, toll-like receptor 4.

To determine whether TLR4 is important in LPS-induced tumor cell metastasis and EMT, we used adeno-associated virus to express TLR4 in HepG2 cells [See, Additional file 1, Figure S2A, B], then pretreated vec-HepG2 and TLR4-HepG2 with LPS. The scratch wound-healing and Matrigel invasion assay showed that overexpression of TLR4 enhanced LPS-induced motility and invasion of HepG2, accompanied by EMT (a decreased expression in epithelial cell markers, such as E-cadherin and β-catenin, and an increased expression of mesenchymal cell markers, such as Vimentin, N-cadherin, and α-SMA. (Figure 4A, B, C). We then knocked down TLR4 in SMMC-7721 cells with siRNA [See Additional file 1, Figure S 2C, D]; reduction of TLR4 expression in SMMC- 7721 cells reversed the changes in cell motility, invasion and EMT induced by LPS. (Figure 4A, B, C). We next examined whether TLR4 expression influenced LPS-induced tumor cell metastasis *in vivo*. Transfected cells were stimulated by LPS for 48 hours *in vitro *and then injected intrasplenically into athymic nude mice. The nude mice splenic vein metastasis models showed that mice injected with LPS-treated TLR4-HepG2 cells had more metastatic nodules on the liver surface than mice with LPS-treated vec-HepG2 cells (Figure 4D, E, P < 0.05). Reduced TLR4 expression in SMMC-7221 resulted in fewer liver surface metastatic nodules in mice compared to mice injected with LPS-treated SMMC-7721 cells (Figure 4D, F, P < 0.05). These results indicate that LPS-induced cell metastasis and EMT in HCC cells are TLR4 dependent.

### LPS-induced EMT involves NF-κB activation and increase of Snail expression

**Figure 5:**
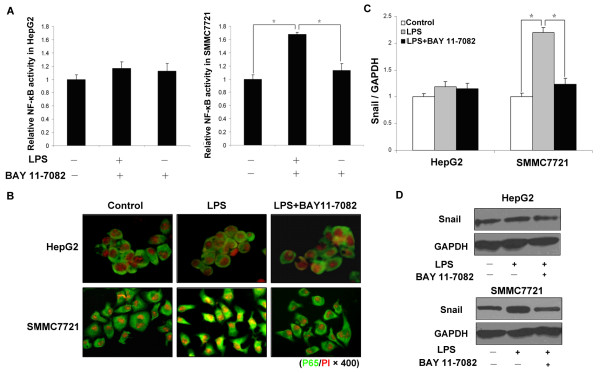
**LPS/TLR4 induces cells EMT through activation of NF-κB**. **(A**) A dual luciferase assay was performed 48 hours after adding LPS. Relative NF-kB luciferase activity, normalized to Renilla luciferase activity, was expressed relative to that of control, set at 1.0. LPS resulted in a significant increase of luciferase activity in SMMC-7721 cells, but had no effect in HepG2 cells; NF-κB inhibitors suppress NF-κB activation in SMMC-7721 cells (*P < 0.05). (**B**) Immunofluoresence was used to evaluate the NF-κB p65 binding activity of pretreated HepG2 and SMMC-7721 cells. The p65 subunit of NF-κB in the cytosol was stained green and nuclei were stained red with PI (× 400). (**C**) and (**D**) qPCR and western-blot were used to detect Snail expression in pretreated HepG2 and SMMC-7721 cells. The results showed that LPS upregulated Snail expression in SMMC-7721 cells, but not in HepG2 cells, NF-κB inhibitors suppress Snail expression in SMMC-7721 cells (* *P *< 0.05). EMT, epithelial-mesenchymal transition; HCC, hepatocellular carcinoma; LPS, lipopolysaccharide; NF-κB nuclear factor kappa B; TLR4, toll-like receptor 4.

LPS-induced TLR4 signaling has been shown to activate the NF-κB signaling pathway in various cell types [8,24]. To clarify the relationship between NF-κB activation and the enhanced invasion induced by LPS, we assessed NF-κB activation induced by LPS in HepG2 and SMMC-7721 cells with pNF-κB-Luc reporter vector. As shown in Figure 5A, LPS treatment resulted in a significant increase of luciferase activity in SMMC-7721 cells (*P *< 0.05), but had no effect in HepG2 cells (*P *> 0.05). Translocation of the p65 subunit of NF-κB to cell nuclei and NF-κB p65 binding activity were evaluated by immunofluoresence; after LPS treatment, p65 subunit translocation significantly increased only in SMMC-7721 cells, whereas no changes were observed in HepG2 cells (Figure 5B). BAY 11-7082 can inhibit phosphorylation of IκBα and subsequent NF-κB nuclear translocation. The results showed that 10 μM BAY 11-7082 inhibited NF-κB activity induced by LPS in SMMC-7721 cells significantly (*P *< 0.05) but had no effect in HepG2 cells (*P *> 0.05), which indicated that LPS-induced NF-κB activation is TLR4 dependent.

Margit AH *et al. *had shown that NF-κB is a pivotal regulator of the EMT process during distinct steps of breast cancer progression [25]. In our study, we examined how NF-κB activation induced by LPS/TLR4 enhanced expression of transcription factor Snail (Figure 5C, D), a major inducer of EMT. Expression of Snail in SMMC-7721 cells was sufficient to induce EMT as measured by the down modulation of E-cadherin transcript and protein [See Additional file 1, Figure S3]. Increased expression of Snail could also be blocked by treatment with BAY 11-7082 (Figure 5C, D). Together with the data presented in Figure 5A, these results showed that treatment with LPS activated NF-κB depending on TLR4 signaling which subsequently caused an increase in Snail and led to EMT and invasion in HCC cells.

### Association of TLR4 expression with clinicopathologic characteristics and prognosis of HCC

**Figure 6:**
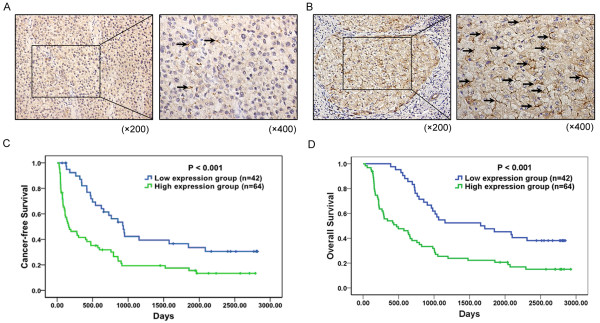
**Immunohistochemistry of TLR4 expression in HCC tissues and its prognostic implication**. (**A**) and (**B**) The membranous expression of TLR4 was seen to be low in 42/106 of HCC tissues (A), and high in 64/106 (B). Original magnification left × 200, right × 400. (**C**) Estimated cancer-free survival according to the expression of TLR4 in 106 cases of HCCs (the Kaplan-Meier method). Log-rank test shows that HCC patients in the high TLR4 expression group have poorer cancer-free survival than those in the low TLR4 expression group (*P *< 0.001). (**D**) Overall survival was analyzed in the same cohort of HCC patients and the results showed that HCC patients in the high TLR4 expression group also have poorer overall survival than those in the low TLR4 expression group (*P *< 0.001). HCC, hepatocellular carcinoma; TLR4, toll-like receptor 4.

In HCC tissues, a total of 101 of 106 (86%) samples showed positive TLR4 expression (Figure 6A, B). The TLR4 expression levels were found to be significantly higher in HCC tissues with cirrhosis (*P *= 0.018), tumor size (*P *= 0.030), margin (*P *= 0.019), vascular invasion (*P *= 0.019), portal vein thrombosis (*P *= 0.014), and UICC T stage (*P *< 0.001) (Table 1). According to the immunohistochemistry results, all 106 HCC patients were divided into two groups: the high expression group (n = 64) and low expression group (n = 42) (Figure 6A, B). Most of the HCC patients within the high expression group had tumor thrombus, and the IHC results showed that TLR4 expression in tumor thrombus was higher than in normal liver tissue [See Additional file 1, Figure S4]. In addition, HCC patients within the high expression group had either worse cancer-free survival (median cancer-free survival time, 576.83 days versus 1,151.45 days, *P *< 0.001; Figure 6C) or worse overall survival (median survival time, 870.45 days versus 1,689.43 days, *P *< 0.001; Figure 6D) than those within the low expression group.

**Table 1:** Correlations between TLR4 expression and clinicopathologic variables of HCC.

Clinicopathologic Parameters	Number	TLR4 expression levels		*P*-Value
			
		Low	High	
**Age (years)**				
≤60	83	36	47	0.134
>60	23	6	17	
**Gender**				
Male	88	36	52	0.546
Female	18	6	12	
**Cirrhosis**				
Absence	42	17	35	0.018*
Presence	64	35	29	
**AFP (ng/mL)**				
≤20	24	11	13	0.479
>20	82	31	51	
**HBsAg**				
Negative	15	7	9	0.714
Positive	90	35	55	
**Tumor size (cm)**				
≤5	35	19	16	0.030*
>5	71	23	48	
**Tumor margin**				
Clear	34	19	15	0.019*
Invovled	72	23	49	
**Satellite lesion**				
Absence	77	32	45	0.507
Presence	29	10	19	
**Vascular invasion**				
Absence	23	14	9	0.019*
Presence	83	28	55	
**Portal vein thrombosis**				
Absence	74	35	39	0.014*
Presence	32	7	25	
**UICC T stage**				
T1	16	13	3	<0.001*
T2	60	26	34	
T3	30	3	27	

(*P <0.05)

## Discussion

To study the key roles of TLR4 in LPS-induced EMT of HCC cells, we selected four different HCC cells (HepG2, Huh7, Hep3B, and SMMC-7721) and carefully evaluated the direct effect of LPS on the invasive potential of these cells. We demonstrated that LPS could significantly enhance the invasive potential and induce EMT of HCC cells with higher expression of TLR4, which was confirmed to be in a TLR4 dependent manner. Further studies showed that LPS could directly activate NF-κB signaling through TLR4 in HCC cells. Importantly, blocking NF-κB signaling significantly inhibited Snail expression and inhibited EMT occurrence. Finally, we revealed that high expression of TLR4 in HCC tissues was strongly associated with both poor cancer-free survival and overall survival. Our results indicate that TLR4 is necessary for LPS-induced EMT and metastasis in HCC cells, which increases our understanding of the pathogenesis and provides clues for developing new strategies against LPS-related metastasis of HCC.

This study provides the first evidence that LPS-induced metastasis and EMT of HCC cells is TLR4 dependent. EMT is a key event in the metastasis of tumors from epithelial cells. An imaging study *in vivo *has shown that carcinoma cells migrate from mouse primary tumors through a process of EMT and that this process is dependent on an inflammatory microenvironment [26]. Recently, the new finding that TNF-α induces EMT in MCF-7 breast cancer cells reinforced the connection between inflammation and EMT [27]. In HCC patients, the level of endotoxin in the portal and peripheral veins has been determined to be higher, which results in a high level of LPS in the portal vein. In this study, four different human HCC cell lines (HepG2, Huh7, Hep3B, and SMMC-7721) were tested in the scratch wound-healing and Matrigel invasion assay. Our results showed that not all tumor cell lines responded *in vitro *to LPS, the motility and invasion abilities of SMMC-7721 cells were enhanced significantly, and the data of the splenic vein metastasis assay *in vivo *were consistent with this. However, Liu *et al. *have inferred that LPS may be ignored by immune systems and may directly promote the invasion abilities of HepG2 and HepG2.2.15 cells [9]. Our experiment showed that LPS had little effect on HepG2 cells; the different experimental methods may have led to the inconsistent results.

Administration of LPS can induce EMT of human IBECs, which makes tumor cells undergo a phenotypic change to produce bipolar cells with a fibroblastic morphology. In these four HCC cells, LPS induced EMT in SMMC-7721 cells but not the others, and SMMC-7721 cells constitutively expressed a high level of TLR4, which might be associated with LPS-induced metastasis and EMT of HCC cells. When we used adeno-associated virus to express TLR4 in HepG2 cells or knocked down TLR4 in SMMC-7721 cells with siRNA, the metastasis and EMT of HCC cells were changed by being treated with LPS. All of the above results highlight a direct link between LPS and tumor cell metastasis. The effect of LPS is possible because SMMC-7721 cells constitutively express TLR-4. Not all tumor cells are positive for TLR-4, and this could depend on the tissue from which the tumor cells originated. Molteni *et al. *analyzed the responses of tumor cell lines from different origins (melanoma, ovarian carcinoma, neuroblastoma) to LPS *in vitro; *the results showed that only melanoma cells significantly increased cell adhesion, when triggered with LPS and these effects were associated with the constitutive expression of TLR-4 [28]. Different HCC cell lines also have different expression of TLR4, and LPS-induced EMT in cells is in a TLR4 dependent manner as well. However, notably, HCC development is a multifactorial and complicated process, which has a close association with various risk factors, such as viral infection, alcohol consumption, steatosis, toxic substances and inflammation. In this process, LPS is an important EMT inducer but not the only one. Many gene alterations and cytokines also could induce EMT and promote metastasis [29]. Therefore, although HCC cells with low expression or even a lack of TLR4 are not susceptible to LPS, they might perform EMT induced by other TLR4-independent mechanisms.

NF-κB activation is well known to play an important role in responses to LPS via TLR-4 [8,24]. Previous studies have shown that LPS may be able to induce NF-κB activation in colon and pancreatic cancer cells [30,31], which strongly suggests that LPS can act not only on immune cells but also on some cancer cells from epithelial cells. In the current study, we found that ligation of LPS and TLR4 could activate the NF-κB pathway in SMMC-7721 cells, but not in HepG2 cells. Furthermore, activation of NF-κB is involved in the invasion and metastasis of HCC. Many studies have shown that NF-κB is a key regulator of Snail expression in cancer cell lines and metastatic tumor samples [32-35]. In our study, activation of NF-κB in SMMC-7721 cells up-regulated Snail expression, which was a prerequisite for these cells to undergo an EMT toward an invasive, metastatic tumor phenotype. Snail is identified as a transcription factor in the control of EMT, and expression of Snail represses expression of E-cadherin and induces EMT in breast cancer cells, indicating that Snail plays a fundamental role in EMT [36,37]. To confirm the contribution of NF-κB activation to the enhanced invasive ability and EMT, we used BAY-11-7082 to inhibit NF-κB activation in SMMC-7721 cells, and the inhibitor not only reduced NF-κB activation, but also down-regulated Snail expression. Our data strongly suggest that activation of the NF-κB pathway by LPS is TLR4-dependent and increases Snail expression to induce EMT.

TLR4 expression has been described in different human tumors [10,12-16]. Gonzalez-Reyes *et al. *have showed that TLR4 expression in breast carcinomas was associated with an increased incidence of metastasis and has prognostic significance [14]. Cammarota *et al. *analyzed 116 tissue samples from patients with different stages of colorectal disease and found that adenocarcinoma patients with higher TLR-4 expression in the stromal compartment had a significantly increased risk of disease progression and relapsed significantly earlier than those with lower expression levels [38]. Interestingly, analysis of the association of TLR4 expression and the clinicopathological characteristics of 106 HCC patients reveals that TLR4 expression is significantly correlated with margin, vascular invasion, portal vein thrombosis, and so on, which are widely accepted markers for metastasis and poor prognosis of HCC [39,40]. The Kaplan-Meier analysis shows that the HCC patients with high TLR4 expression in general had a shorter cancer-free interval and a worse overall survival than those with low expression, suggesting that TLR4 may be a useful biomarker of HCC metastasis and recurrence.

In conclusion, our study has shown for the first time that LPS-induced EMT and the invasive potential of HCC cells is in a TLR4 dependent manner and that activation of the NF-κB-Snail regulatory axis may function as a therapeutic target. Furthermore, we have demonstrated that high expression of TLR4 in HCC tissues significantly correlates with metastasis and recurrence of HCC. Our data suggest TLR4 as a novel prognostic marker and a potential therapeutic target for LPS-induced EMT and metastasis of HCC.

## Conclusions

Our results indicate that TLR4 signaling is required for LPS-induced EMT, tumor cell invasion and metastasis, which provide molecular insights for LPS-related pathogenesis and a basis for developing new strategies against metastasis in HCC.

## Abbreviations

ANOVA: analysis of variance; α-SMA: α-smooth muscle actin; (D)MEM: (Dulbecco's) modified Eagle's medium; EDTA: ethylenediamine tetraacetic acid; EMT: epithelial to mesenchymal transition; FBS: fetal bovine serum; FITC: fluorescein isothiocyanate; HCC: hepatocellular carcinoma; IBECs: intrahepatic biliary epithelial cells; IHC: immunohistochemisty; LPS: lipopolysaccharide; NF-κB; nuclear factor kappa B; qPCR: quantitative real-time polymerase chain reaction; SEM: standard error of the mean; TLR4: toll-like receptor 4.

## Competing interests

The authors declare that they have no competing interests.

## Authors' contributions

YYJ, ZPH, KS and LXW participated in the design and performance of the study. SSZ, JH and YL provided clinical data and performed statistical analysis. RL, LG, XZ and QDZ carried out cell culture, molecular genetic studies and the mouse experiment. MCW and LXW conceived of the study and participated in its design and coordination. The manuscript was drafted by YYJ, ZPH and KS, and reviewed by all authors. All authors approved the final version of the manuscript to be published.

## Pre-publication history

The pre-publication history for this paper can be accessed here:

http://www.biomedcentral.com/1741-7015/10/98/prepub

## Supplementary Material

Additional file 1 **Supplemental figures and tables**. **Figure S1. MHCC97-H cells with high expression of TLR4 were induced metastasis and EMT by LPS**. (A) FACS analysis for TLR4 expression in MHCC97-H cell line untreated or treated with LPS (10 μg/ml) for 48 hours. (B) The invasiveness of MHCC97-H cells pretreated with LPS (10 μg/ml) for 48 hours was determined by Transwell assay (× 200, *P < 0.05). (C) qPCR was used to detected changes in the expression of EMT genes in MHCC97-H cells treated with LPS. Results presented represent mean of triplicate experiments ± SEM (*P < 0.05). (D) Immunofluorescent staining of E-cadherin and Vimentin was performed in MHCC97-H cells that were either untreated or treated with LPS, nuclei were counterstained with DAPI (× 400). **Figure S2. Upregulation of TLR4 expression in HepG2 cells and downregulation of TLR4 expreesion in SMMC-7721 cells**. (A) and (B) Adeno-associated virus was used to express TLR4 in HepG2 cells. TLR4 mRNA expression was detceted by qPCR (A, *P < 0.05) and protein expression was detected by western-blot (B). (C) and (D) siRNA was used to knock down TLR4 expression in SMMC-7721 cells. TLR4 mRNA expression was detceted by qPCR (C, *P < 0.05) and protein expression was detceted by western-blot (D). **Figure S3. Upregulation of Snail induced EMT in SMMC-7721 cells**. Adeno-associated virus was used to express Snail in SMMC-7721 cells. E-cad mRNA expression was detected by qPCR (A*P < 0.05), and E-cad protein expression was evaluated by western-blot (B). The data shown in A and B are from one representative experiment of three performed. **Figure S4. High expression of TLR4 in HCC thrombus**. (A) H & E staining was performed to show thrombus in HCC tissues (× 200). (B) Immunohistochemistry was performed to show TLR4 expression in HCC thrombus as well as surrounding normal liver tissue (× 200), (a) HCC thrombus, original magnification × 400 (b) normal liver tissue, original magnification ×400. BV, blood vessels; T, thrombus; N, normal liver tissue. **Table S1**. Description: Sequence of the oligonucleotides for real-time PCR assays Click here for file
