# Supplementary material for: Toll-like receptor 4 signaling promotes epithelial-mesenchymal transition in human hepatocellular carcinoma induced by lipopolysaccharide
Source: BMC Med. 2012 Aug 31;10:98. doi: 10.1186/1741-7015-10-98 (PMC3482562; doi:10.1186/1741-7015-10-98)
Supplement: Additional file 1 — Supplemental figures and tables. Figure S1. MHCC97-H cells with high expression of TLR4 were induced metastasis and EMT by LPS. (A) FACS analysis for TLR4 expression in MHCC97-H cell line untreated or treated with LPS (10 μg/ml) for 48 hours. (B) The invasiveness of MHCC97-H cells pretreated with LPS (10 μg/ml) for 48 hours was determined by Transwell assay (× 200, *P < 0.05). (C) qPCR was used to detected changes in the expression of EMT genes in MHCC97-H cells treated with LPS. Results presented represent mean of triplicate experiments ± SEM (*P < 0.05). (D) Immunofluorescent staining of E-cadherin and Vimentin was performed in MHCC97-H cells that were either untreated or treated with LPS, nuclei were counterstained with DAPI (× 400). Figure S2. Upregulation of TLR4 expression in HepG2 cells and downregulation of TLR4 expreesion in SMMC-7721 cells. (A) and (B) Adeno-associated virus was used to express TLR4 in HepG2 cells. TLR4 mRNA expression was detceted by qPCR (A, *P < 0.05) and protein expression was detected by western-blot (B). (C) and (D) siRNA was used to knock down TLR4 expression in SMMC-7721 cells. TLR4 mRNA expression was detceted by qPCR (C, *P < 0.05) and protein expression was detceted by western-blot (D). Figure S3. Upregulation of Snail induced EMT in SMMC-7721 cells. Adeno-associated virus was used to express Snail in SMMC-7721 cells. E-cad mRNA expression was detected by qPCR (A*P < 0.05), and E-cad protein expression was evaluated by western-blot (B). The data shown in A and B are from one representative experiment of three performed. Figure S4. High expression of TLR4 in HCC thrombus. (A) H & E staining was performed to show thrombus in HCC tissues (× 200). (B) Immunohistochemistry was performed to show TLR4 expression in HCC thrombus as well as surrounding normal liver tissue (× 200), (a) HCC thrombus, original magnification × 400 (b) normal liver tissue, original magnification ×400. BV, blood vessels; T, thrombus; N, normal liver tissue. Ta [file 1741-7015-10-98-S1.PDF]

:

Figure S1

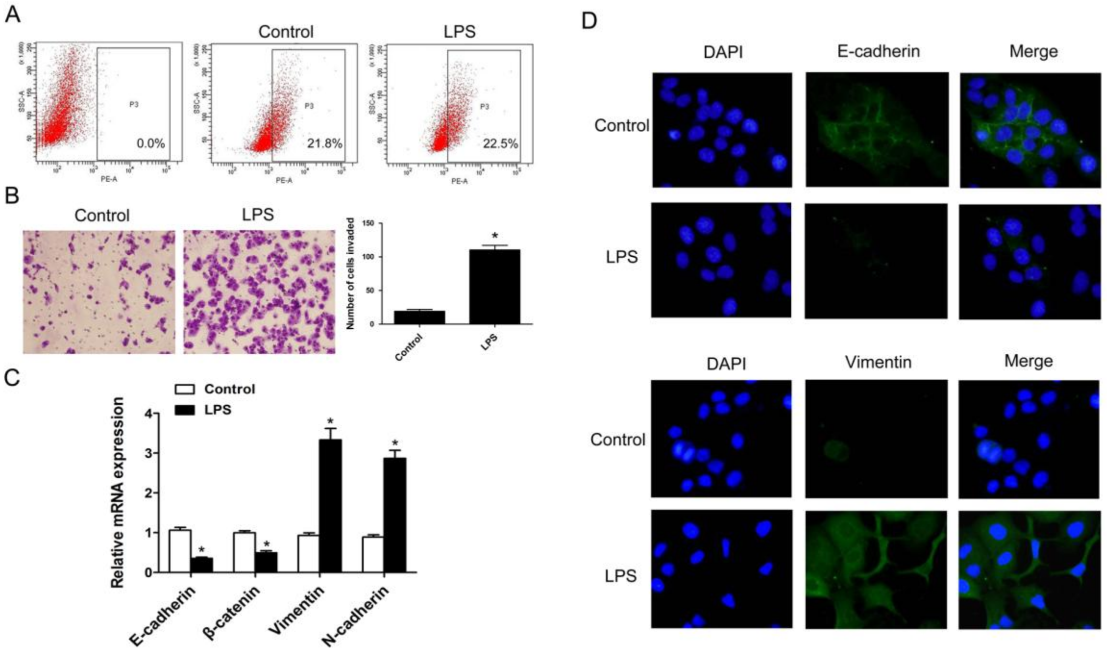

Figure S2

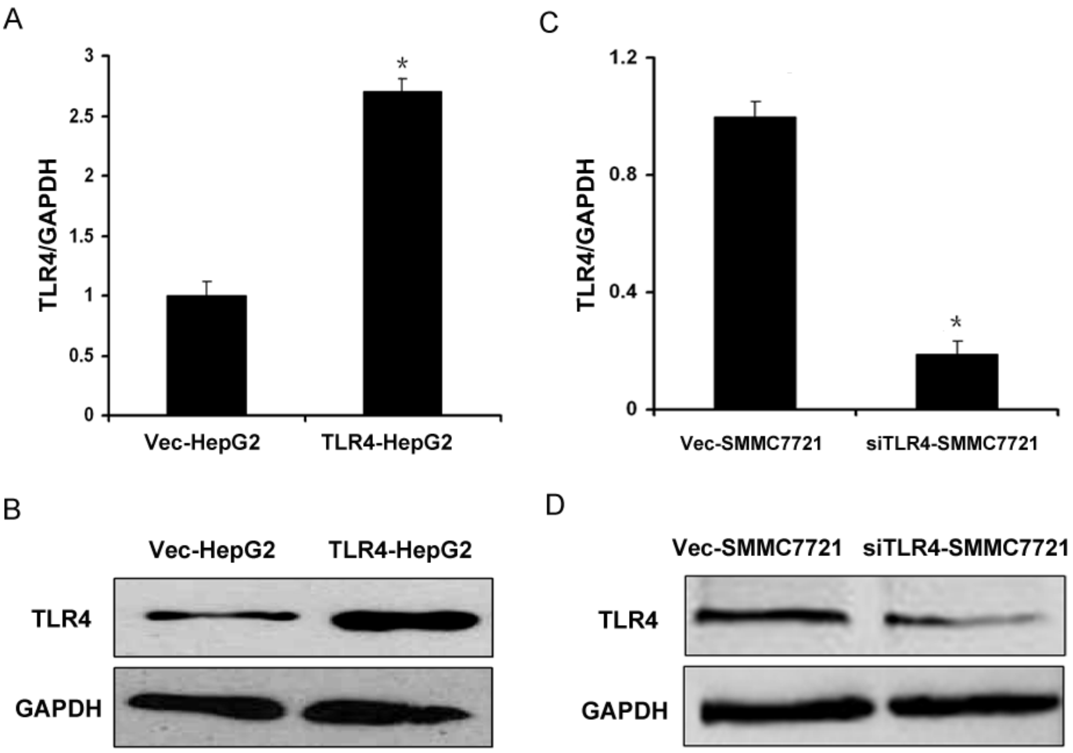

Figure S3

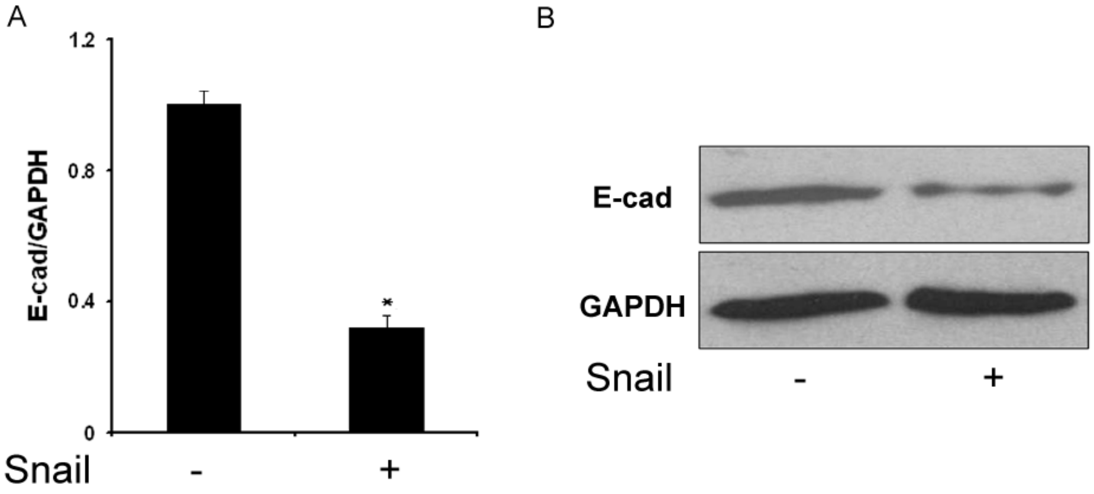

Figure S4

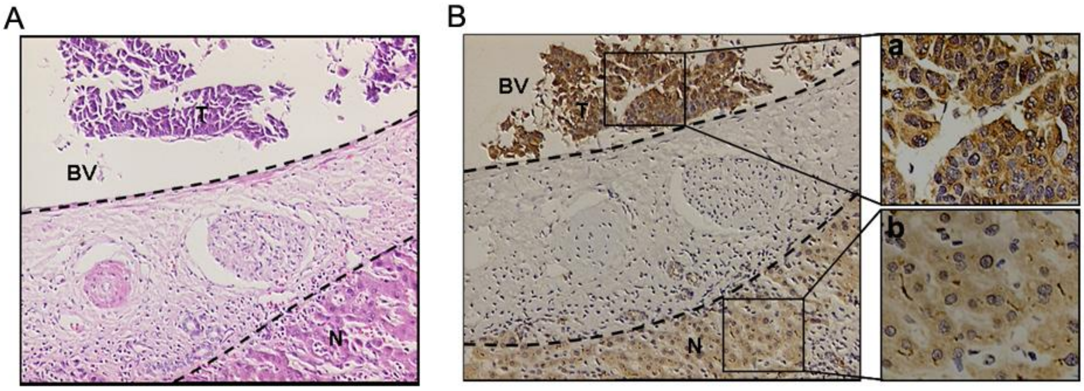

Table S1

Additional Table S1. Sequence of the oligonucleotides for real-time PCR assays

Gene		Sequence (5' → 3')
E-cadherin	F	TGAAGGTGACAGAGCCTCTGGA
	R	TGGGTGAATTCGGGCTTGTT
Vimentin	F	TGGCCGACGCCATCAACACC
	R	CACCTCGACGCGGGCTTTGT
N-cadherin	F	GCGCGTGAAGGTTTGCCAGTG
	R	CCGGCGTTTCATCCATACCACAA
α-SMA	F	GGTCCATGTCCGCGTCCCACTAG
	R	CGCCCCACGCCCTGTTTCTT
β-catenin	F	AGCCGACACCAAGAAGCAGAGATG
	R	CGGCGCTGGGTATCCTGATGT
GAPDH	F	AGAAGGCTGGGGCTCATTTG
	R	AGGGGCCATCCACAGTCTTC
